# Management of cardiac drugs in a critical care setting

**DOI:** 10.1186/cc11122

**Published:** 2012-03-20

**Authors:** M Mallick, J Walkington, A Gratrix, R Pretorius

**Affiliations:** 1 Hull Royal Infirmary, Hull, UK; 2 York Teaching Hospital, York, UK

## Introduction

ICU admissions may lead to discontinuation of longstanding evidence-based therapies. A recent study demonstrated how such medications have been discontinued for patients even after their ICU stay [1]. Evidence has shown the beneficial role of β-blockers in the perioperative period [2], and roles for other drugs such as angiotensin-converting enzyme inhibitors (ACE-I) and statins have been demonstrated. The aim of the current study was to examine 30-day mortality and complication rates in the critical care population who were on cardiac medications and did not receive these medications during their ICU stay.

## Methods

We looked retrospectively at the last 80 patients admitted to the ICU or HDU in York, 2011. The patients' case notes were examined to assess if they were on cardiac medications and if those drugs were omitted during their admission. The cardiac medications assessed were β-blockers, ACE-I and statins. We also reviewed any cardiac complications incurred during their stay, alongside 30-day mortality.

## Results

A total of 29.6% of patients on β-blockers received them, whilst 67.8% did not. Complication and mortality rates for medications given versus not given were 12.5% versus 68.4% and 0% versus 42.1% (*P *= 0.003 and *P = *0.007) respectively. A total of 17.6% of patients on ACE-I received them, whilst 82.3% did not. Complication and mortality rates for medications given versus not given were 0% versus 9.0% and 0% versus 35.7% (*P *= 0.004 and *P *= 0.055) respectively. A total of 31.6% of patients on statins received them, whilst 68.4% did not. Complication and mortality rates for medications given versus not given were 25.0% versus 42.3% and 8.3% versus 38.5% (*P *= 0.256 and *P = *0.02 respectively). The global complication and mortality rates for medications given versus not given were 28% versus 55.2% and 11.5% versus 51.7% (*P *= 0.0648 and *P *= 0.0039) respectively. Omission of β-blockers resulted in significantly higher complication and mortality rates. Omission of ACE-I resulted in higher complication rates and of statins in higher mortality rates. Omission of cardiac medications resulted in a significantly higher mortality rate.

## Conclusion

The study does highlight a trend associated with patients who are on medications who do not receive them to either develop higher complication rates or higher mortality rates or both. Further research involving larger numbers is required to produce validated opinions.

## References

[B1] Bell CM JAMA 2011 306 840 847 10.1001/jama.2011.1206 21862745

[B2] 2009 ACCF/AHA focused update on preoperative beta blockade: a report of the American College of Cardiology Foundation/American Heart Association task force on practice guidelines Circulation 2009 120 2123 2151 19884474 10.1161/CIRCULATIONAHA.109.192689

